# Vitreous Hemorrhage Following Blunt Ocular Trauma by a Cat’s Forepaw: A Case Report and Literature Review

**DOI:** 10.7759/cureus.89324

**Published:** 2025-08-04

**Authors:** Daisuke Hasegawa, Yui Nishijima, Tatsuya Mimura

**Affiliations:** 1 Department of Ophthalmology, Tsurumi University School of Dental Medicine, Yokohama, JPN; 2 Department of Ophthalmology, Keio University School of Medicine, Tokyo, JPN; 3 Department of Ophthalmology, Teikyo University School of Medicine, Tokyo, JPN

**Keywords:** blunt eye injury, cat, forepaw, vitrectomy, vitreous hemorrhage

## Abstract

Traumatic vitreous hemorrhage is most commonly associated with sports injuries or accidental falls. We report an exceptionally rare case of vitreous hemorrhage presumed to result from blunt trauma inflicted by the forepaw of a domestic cat. A 50-year-old man presented with blurred vision and ocular pain in his right eye after being struck by his pet cat. Initial examination revealed an uncorrected visual acuity of 20/200 in the right eye, which improved to 20/25 with correction. Intraocular pressure (IOP) was measured at 16 mmHg in the right eye and 17 mmHg in the left. Slit-lamp and fundoscopic examinations revealed subconjunctival hemorrhage, subretinal hemorrhage, and vitreous hemorrhage in the right eye, with no signs of iritis or other indications of cat scratch disease. Based on these findings, the condition was diagnosed as an intraocular hemorrhage due to blunt ocular trauma. Conservative management was initially pursued; however, because of persistent vitreous opacity, cataract progression, and elevated intraocular pressure (30 mmHg), combined phacoemulsification and pars plana vitrectomy were performed seven months post-injury. Postoperative recovery was favorable, with corrected visual acuity improving to 20/16 and intraocular pressure stabilizing between 14 and 16 mmHg.

A comprehensive review of the literature identified 11 previously reported cases of ocular trauma caused by domestic cats, documented in seven separate reports. All involved penetrating globe injuries from cat claws, and no cases of blunt trauma caused by a cat’s forepaw were found. This case, therefore, represents, to our knowledge, the first report of such a mechanism. It highlights that even seemingly benign interactions with domestic animals can lead to significant ocular injury. Although uncommon, this case suggests that even benign interactions with domestic animals may pose a risk for ocular trauma, and clinicians should remain vigilant in assessing such presentations.

## Introduction

Traumatic vitreous hemorrhage is a relatively common ocular injury, typically associated with high-energy blunt trauma to the globe resulting from sports injuries, traffic accidents, falls, or physical assault [1]. Such injuries can occur across a wide age range, from young individuals to the elderly, and the visual prognosis varies greatly depending on the cause of trauma, the circumstances of the injury, and the management provided immediately after the injury. Vitreous hemorrhage rarely occurs in isolation; it is often accompanied by damage to other intraocular structures, such as retinal tears, retinal detachment, choroidal hemorrhage, intraocular pressure (IOP) abnormalities, or cataract, all of which influence treatment decisions and the recovery of visual function.

Traumatic vitreous hemorrhage has been extensively described in several recent review articles [2,3]. It is a relatively common ocular emergency, particularly affecting young men, and accounts for approximately 12%-18% of all cases of vitreous hemorrhage. The most frequent etiologies include sports injuries, physical assaults, and accidental falls, with blunt trauma being the predominant mechanism. Clinically, patients may present with sudden visual impairment, floaters, or visual haze, depending on the severity and location of the hemorrhage. Initial examination may reveal subconjunctival hemorrhage, elevated intraocular pressure, or signs of posterior segment trauma such as commotio retinae or retinal tears. In cases of dense vitreous hemorrhage that obscures fundus visualization, B-scan ultrasonography remains essential for assessing retinal detachment, intraocular foreign bodies, or posterior scleral rupture. Optical coherence tomography and fluorescein angiography can be useful in follow-up to evaluate macular and retinal status. The differential diagnosis of vitreous hemorrhage includes proliferative diabetic retinopathy, retinal vein occlusion, posterior vitreous detachment with retinal tears, ocular tumors, and Terson syndrome, particularly in non-traumatic cases. Management strategies range from observation and conservative therapy in mild cases to surgical intervention, typically pars plana vitrectomy, for persistent vitreous hemorrhage, elevated intraocular pressure, or associated retinal pathology. Traumatic vitreous hemorrhage may be complicated by secondary glaucoma, cataract progression, retinal detachment, or proliferative vitreoretinopathy (PVR), all of which can have lasting effects on visual outcomes if not promptly addressed. Given these potential complications, timely recognition and appropriate treatment are critical. Animal-inflicted ocular trauma is rare.

Unexpected ocular trauma may also occur in domestic settings during everyday life, with pet-related injuries being a potential cause. Among domestic animals, cats are particularly known to cause facial and ocular injuries due to their sharp claws and swift movements [4-10]. However, vitreous hemorrhage resulting from blunt ocular trauma caused by a strike from a cat’s forepaw is extremely rare, and few cases have been reported that describe the detailed clinical course, management strategies, or visual outcomes.

Here, we report an exceptionally rare case of subretinal and vitreous hemorrhage following blunt ocular trauma caused by a domestic cat’s forepaw strike. The patient ultimately required pars plana vitrectomy combined with cataract surgery due to progressive cataract formation and elevated IOP. We also reviewed the existing literature on ocular trauma caused by cats to contextualize our findings. This case serves as a valuable reminder that routine contact with domestic animals can unexpectedly result in serious ocular injuries and provides important insights into the diagnosis and management of similar trauma.

## Case presentation

A 50-year-old man presented with complaints of blurred vision and ocular pain in his right eye after being struck in the eye by the forepaw of his pet cat. At the initial examination, uncorrected visual acuity was 20/200 in the right eye, improving to 20/25 with a refractive correction of -2.75 diopters (D) sphere and -0.75 D cylinder at 90 degrees. The left eye had uncorrected visual acuity of 20/200, improving to 20/20 with a correction of -2.75 D sphere and -0.50 D cylinder at 20 degrees. IOP was 16 mmHg in the right eye and 17 mmHg in the left eye.

**Figure 1:**
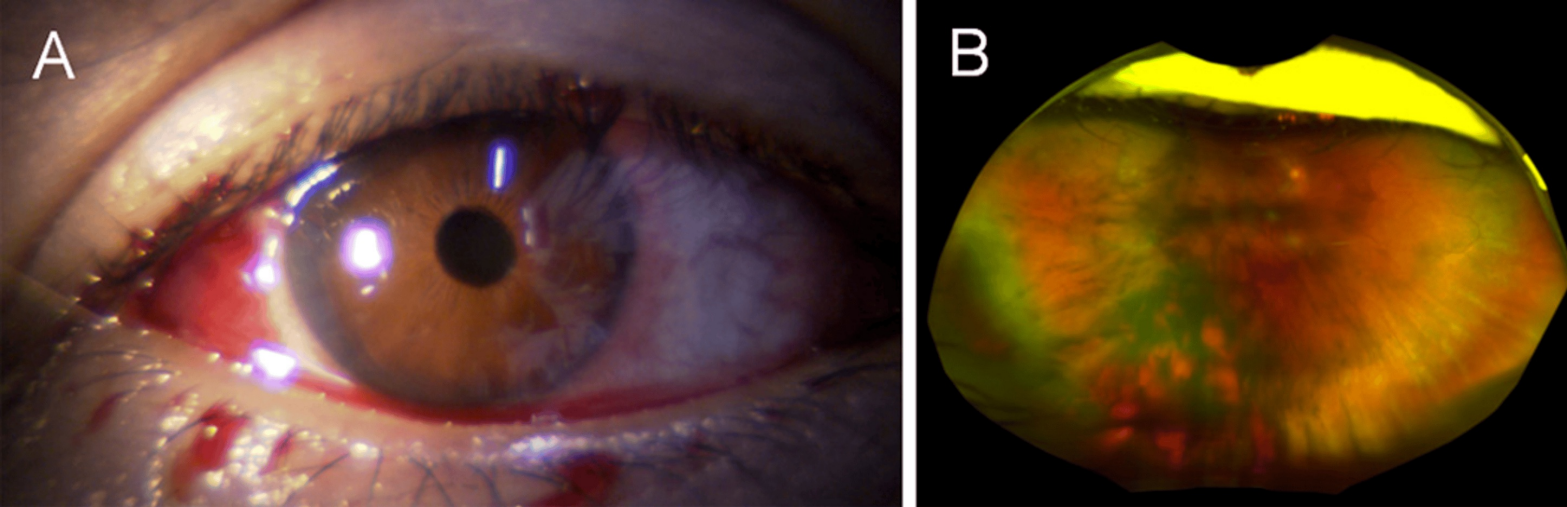
Slit-lamp (A) and fundus (B) photographs of the right eye at initial presentation in a 50-year-old man who sustained blunt trauma from the forepaw of his pet cat

Ophthalmic examination of the right eye revealed subconjunctival hemorrhage, subretinal hemorrhage, and vitreous hemorrhage. There were no signs of iritis characteristic of cat scratch disease. Based on these findings, the patient was diagnosed with intraocular hemorrhage secondary to blunt ocular trauma (Figure 1).

**Figure 2:**
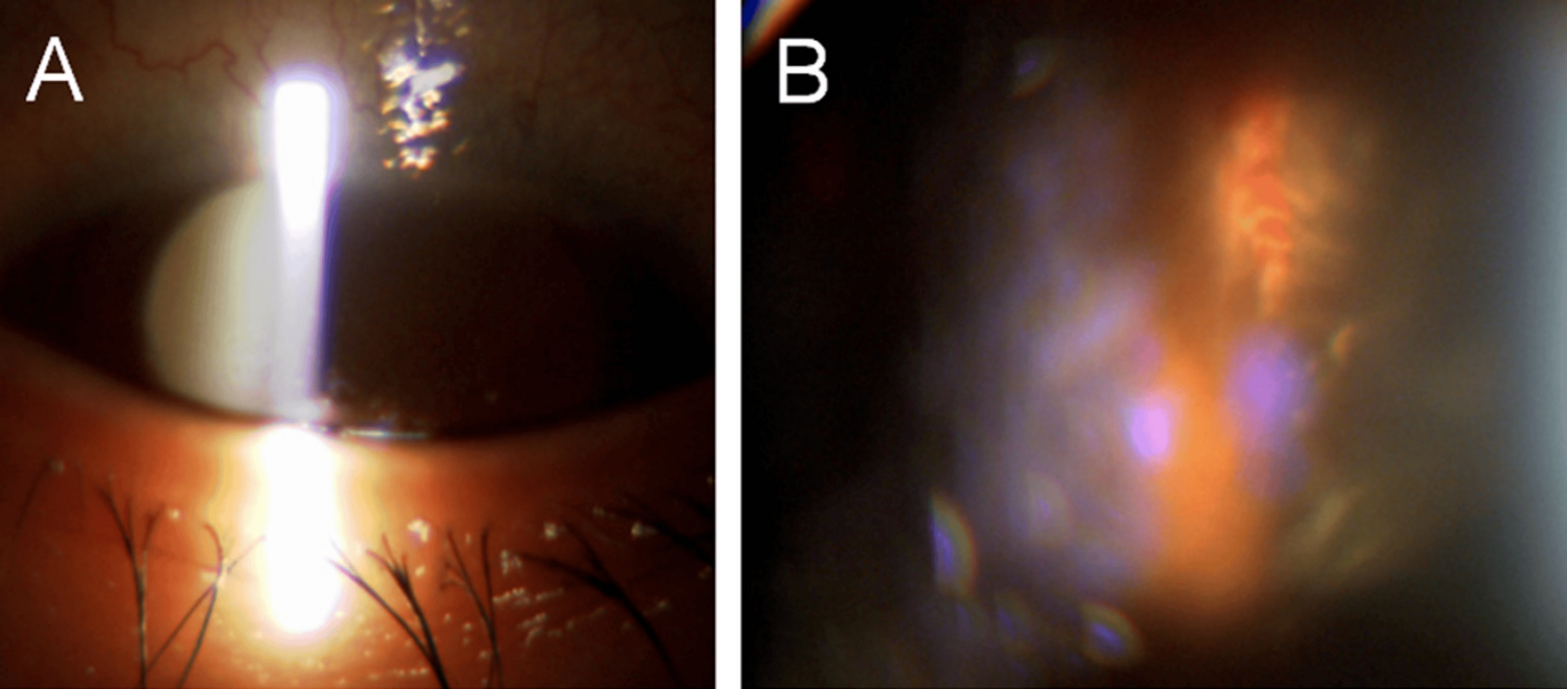
Slit-lamp (A) and fundus (B) photographs of the right eye at seven months after injury

Although conservative treatment was maintained for six months, the patient exhibited persistent vitreous opacity, progressive cataract formation, and elevated intraocular pressure (reaching up to 30 mmHg). Consequently, the visual acuity of the right eye deteriorated to 20/200, and it was no longer correctable with refraction (Figure 2). Subretinal hemorrhages were observed near the superior and inferior retinal arcade vessels, as well as in the peripheral retina immediately after the injury. These hemorrhages had been absorbed by the time of surgery.

**Figure 3:**
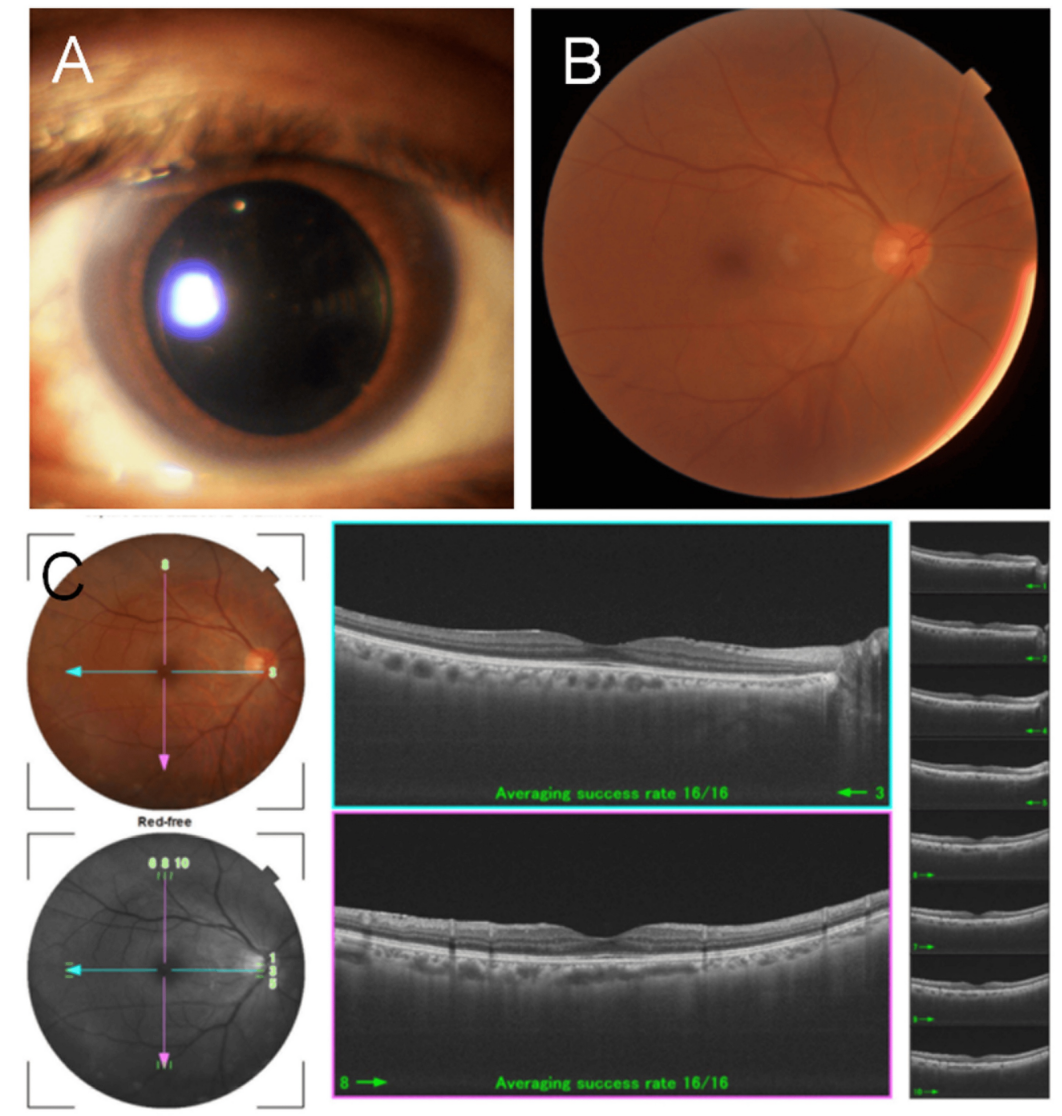
Slit-lamp (A), fundus (B), and optical coherence tomography (C) photographs of the right eye after pars plana vitrectomy and cataract surgery with intraocular lens implantation

Postoperatively, the visual acuity in the right eye improved to 20/25, with best-corrected visual acuity of 20/15 (with refraction of -0.25 D sphere and -0.50 D cylinder at 180 degrees). The IOP remained stable between 14 and 16 mmHg in the right eye (Figure 3).

## Discussion

A literature review was conducted using the PubMed and MEDLINE databases. Various combinations of the following search terms were used: “cat,” “cat scratches,” “cornea,” “corneal laceration,” “ocular trauma,” “Pasteurella,” and “vitreous hemorrhage”. In addition, relevant articles were identified by cross-referencing the citations within the retrieved publications. From each case report, we collected data on patient age, sex, site of ocular injury, treatment methods, and final visual acuity.

The literature review identified 11 cases of corneal laceration caused by cat scratches, reported across seven publications. All cases involved lacerations caused by a cat’s claws; no cases of blunt ocular trauma resulting from a cat’s forepaw strike were found. These cases are summarized in Table 1. The clinical manifestations and severity of ocular injury varied widely among the reported cases. Corneal suturing was performed in five of the 10 cases, and pars plana vitrectomy was performed in two cases. Among the seven cases in which final visual acuity was documented, four achieved a final visual acuity of 20/20 or better.

**Table 1:** Summary of cases of ocular blunt trauma caused by cat claw or forepaw. Age: years; M: male; F: female; USA: United States of America; RD: retinal detachment; VH: vitreous hemorrhage; CRAO: central retinal artery occlusion; AC: anterior chamber; PPV: pars plana vitrectomy; IOL: intraocular lens; BCVA: best-corrected visual acuity; HM: hand motion

Number	Age/sex (onset age)	Country	Cat’s claw or paw	Ocular involvement	Main therapy	Final BCVA	Journal	Year	Author
1	6/M	France	Claw	Hypopyon/iris prolapse/corneal laceration	AC washout/repositioning of the iris/corneal suture/topical, intracameral, and oral antibiotics	Not reported	Bulletin des Societes d'Ophtalmologie de France	1989	Algan et al. [4]
2	33/F	Japan	Claw	Endophthalmitis/RD	PPV	20/200	Ophthalmic Surgery, Lasers and Imaging	1999	Doi et al. [5]
3	10/F	USA	Claw	Corneal laceration/hypopyon	Corneal suture/topical antibiotics/intravitreal antibiotics	20/80	Journal of Pediatric Ophthalmology and Strabismus	2002	Sylvester et al. [6]
4	3/M	USA	Claw	Corneal laceration/anterior capsule tear/cataract	Topical antibiotics/PPV/lensectomy	Unmeasurable	Journal of Pediatric Ophthalmology and Strabismus	2002	Sylvester et al. [6]
5	42/M	Italy	Claw	VH	Oral antibiotics	20/20	International Ophthalmology	2011	Pinna et al. [7]
6	66/M	Italy	Claw	CRAO	Oral antibiotics	HM	International Ophthalmology	2011	Pinna et al. [7]
7	35/F	Australia	Claw	Corneal laceration	Corneal suture/topical and oral antibiotics	6/6	Clinical and Experimental Ophthalmology	2012	Chang et al. [8]
8	3/M	Australia	Claw	Corneal laceration/iris prolapse/cataract	Corneal suture/topical and oral antibiotics/extracapsular cataract extraction and IOL implantation	Unmeasurable	Clinical and Experimental Ophthalmology	2012	Chang et al. [8]
9	7/M (age 2 at the time of injury)	Australia	Claw	Corneal scar	No treatment	6/6	Clinical and Experimental Ophthalmology	2012	Chang et al. [8]
10	32/F	USA	Claw	Corneal laceration/hypotony	Corneal suture	20/15	Canadian Journal of Ophthalmology	2017	Peiris and Khouri [9]
11	10/F	Japan	Claw	Endophthalmitis/RD	PPV/lensectomy	20/20	American Journal of Ophthalmology Case Reports	2020	Mochizuki et al. [10]
12	50/M	Japan	Forepaw	Anterior chamber hemorrhage and VH	PPV/cataract surgery and IOL implantation	20/20	Our case	2025	Hasegawa et al.

This case represents an extremely rare instance in which blunt trauma caused by the forepaw of a domestic cat resulted in vitreous hemorrhage and subretinal hemorrhage, followed by progressive cataract formation and elevated IOP, ultimately necessitating vitrectomy and cataract surgery with intraocular lens implantation. In previously reported cases, ocular injuries associated with cats have most commonly involved penetrating trauma, such as corneal lacerations or eyelid injuries caused by claws [4-10]. To our knowledge, detailed descriptions of vitreous hemorrhage secondary to blunt ocular trauma from a cat, as seen in the present case, are exceedingly rare. This case highlights the potential for routine interactions with household cats to result in unexpected and serious intraocular injuries, including vitreous hemorrhage, cataract formation, and elevated intraocular pressure, which may ultimately require surgical intervention.

The vitreous and subretinal hemorrhages observed in this case were likely due to a sudden, forceful impact from the cat’s forepaw, transmitting shock through the ocular wall and leading to rupture of retinal or choroidal vessels. In general, the visual prognosis in blunt ocular trauma with vitreous hemorrhage is largely determined by the presence or absence of associated retinal tears or detachment. Fortunately, in this case, no retinal tear or detachment was detected, which likely contributed to the favorable visual outcome.

As time progressed, the patient developed cataract and elevated IOP. Traumatic cataract typically results from structural damage to the lens capsule or lens fibers [11], and strong impacts associated with vitreous hemorrhage are known to increase the risk of lens injury. The rise in IOP may have been caused by angle closure due to blood components or inflammatory cells, trabecular meshwork obstruction, or impaired aqueous outflow secondary to breakdown products of intraocular hemorrhage [11]. Gonioscopic examination revealed partial angle closure, supporting this hypothesis. To manage the elevated IOP, topical antiglaucoma medications such as beta-blockers, carbonic anhydrase inhibitors, or prostaglandin analogs can be administered, depending on the underlying mechanism. In some cases, anti-inflammatory agents may also help reduce trabecular meshwork inflammation and improve aqueous outflow. If medical therapy proves insufficient, surgical intervention may be considered to prevent glaucomatous optic nerve damage.

Regarding management, we initially opted for conservative treatment in the hope that the vitreous hemorrhage would clear spontaneously. However, persistent vitreous opacity and progressive cataract eventually necessitated surgical intervention. The therapeutic strategy for traumatic vitreous hemorrhage generally depends on the extent of bleeding, degree of visual impairment, presence of retinal tears or detachment, and other associated complications. Recent reports suggest that early vitrectomy can be beneficial for visual recovery. An international collaborative study by Chauhan et al. demonstrated that patients who underwent “same-day” vitrectomy after ocular trauma achieved the best final visual acuity and had a zero incidence of proliferative vitreoretinopathy (PVR), whereas those with delayed surgery experienced worse visual outcomes and significantly higher risks of PVR and enucleation (p < 0.05) [12]. Nonetheless, in cases without retinal tears or detachment, initial conservative management remains a reasonable option. In the present case, after balancing patient preference and surgical risk, we initially pursued a conservative approach but ultimately achieved excellent visual recovery following cataract surgery with multifocal intraocular lens implantation and vitrectomy. The use of a multifocal intraocular lens also contributed to improved postoperative quality of life, which is noteworthy.

## Conclusions

This case provides several important lessons. First, even interactions with household pets can result in severe, unforeseen ocular injuries, underscoring the need to educate pet owners about potential risks. Particular caution is warranted given the agility and sharp forepaws of cats, especially regarding contact near the face. Second, the case highlights the importance of careful assessment for retinal tears or detachment in traumatic vitreous hemorrhage, as well as the need to determine the optimal timing for intervention based on visual function, IOP trends, and other clinical factors.

In the future, further accumulation of case reports on ocular trauma associated with cats and other pets will be valuable in establishing treatment guidelines. Moreover, public awareness campaigns and patient education efforts are essential to help prevent similar injuries.
